# A Prospective Randomized Controlled Study Comparing Intravenous and Nebulised Dexmedetomidine for Awake Fibreoptic Nasotracheal Intubation

**DOI:** 10.7759/cureus.104003

**Published:** 2026-02-20

**Authors:** Namrata Kaka, Subhash Dahiya, Sweta Singh, Gaurav Sharma, Garima Luthra, Aditi R Singh

**Affiliations:** 1 Anaesthesiology, Critical Care and Pain Medicine, Lala Lajpat Rai Memorial (LLRM) Medical College, Meerut, IND

**Keywords:** airway management, awake fibreoptic intubation, dexmedetomidine, intravenous, intravenous dexmedetomidine, nebulization, nebulized dexmedetomidine, sedation

## Abstract

Background: Awake fiberoptic intubation (AFOI) is the gold standard for anticipated difficult airways but is limited by patient discomfort and hemodynamic stress. Dexmedetomidine may improve tolerance while preserving spontaneous ventilation. We compared intravenous and nebulized dexmedetomidine for AFOI.

Methods: In this prospective, randomized, double-blind trial, 46 American Society of Anesthesiologists (ASA) physical status I-II adults undergoing elective surgery requiring nasotracheal intubation were assigned to two groups. Patients in the nebulization group (Group N) received dexmedetomidine 1 μg/kg, combined with 4 mL of 4% lignocaine, via nebulization for four minutes, along with 20 mL of intravenous normal saline infused over 10 minutes. Patients in the intravenous group (Group I) were administered dexmedetomidine 1 μg/kg diluted in 20 mL normal saline intravenously over 10 minutes, along with 4 mL of 4% lignocaine via nebulization for four minutes. Sedation (Modified Observer's Assessment of Alertness/Sedation scale (OAA/S)), intubation conditions (cough severity, facial grimace), intubation time, hemodynamics, adjunct requirements (propofol dose; spray-as-you-go (SAYGO) lignocaine boluses), discomfort, recall, complications, and willingness to repeat anesthesia were assessed. P <0.05 was considered significant.

Results: Baseline demographics were comparable. Adequate sedation (OAA/S ≤ 3) without propofol occurred in 100% with IV vs 0% with nebulization. Propofol requirement was lower in the intravenous group (0.74 ± 1.14 vs 4.22 ± 1.31 mg/kg; p<0.001). Intubation was faster in the intravenous group (5.22 ± 2.26 vs 8.00 ± 2.02 min; p=0.007). Cough was reduced in the intravenous group (no cough 69.6% vs 0%; severe cough >5 in sequence 0% vs 21.7%). Fewer SAYGO boluses were needed with intravenous (4.00 ± 1.51 vs 5.09 ± 1.88; p=0.04). Hemodynamics were stable and similar overall; one hypotension event occurred in the nebulization group. Postoperative complications were negligible. Discomfort was lower, and willingness to undergo the same technique again was higher with IV dexmedetomidine.

Conclusion: When combined with standardized nebulized lignocaine airway topicalization, intravenous dexmedetomidine 1 μg/kg provided superior sedation quality, better intubating conditions, reduced adjunct use, and shorter intubation time compared with nebulized dexmedetomidine, with comparable safety. Intravenous administration may therefore be preferred for sedation during AFOI.

## Introduction

Awake fiberoptic intubation (AFOI) in a spontaneously breathing patient is widely regarded as the gold standard for managing the difficult airway. However, this technique can be associated with significant nociceptive stimulation, particularly during passage of the endotracheal tube through the nasal passages and larynx. Since fiberoptic intubation (FOI) is often uncomfortable, meticulous preparation is essential. This involves attenuation of airway reflexes, adequate sedation, and anxiolysis, while simultaneously preserving a patent airway and sufficient ventilation [1].

Conscious sedation plays a key role in improving patient tolerance by minimizing awareness, reducing anxiety, and enhancing cooperation during AFOI. It provides optimal intubating conditions even in the presence of abnormal oropharyngeal anatomy or pathology, allowing patients to remain calm and responsive to verbal commands. In contrast, deep sedation carries the risk of airway compromise, hypoventilation, or hypoxia, which may lead to life-threatening consequences. Thus, the greatest challenge during AFOI is to balance adequate sedation and analgesia with maintenance of spontaneous ventilation and oxygenation [2].

Despite its advantages, FOI is not without limitations. Hypoxia, airway secretions, bleeding, local anesthetic allergy, and patient non-cooperation are common concerns. Moreover, because the fiberoptic bronchoscope contains delicate optical elements, even small amounts of secretions or blood can significantly impair visualization. Additionally, the time required to secure the airway via FOI may pose risks in patients with impending hypoxia, making alternative rapid airway management techniques preferable in such scenarios [2].

Patients undergoing AFOI may also experience severe gagging, intense coughing, or even significant pain despite careful administration of local anesthetics [3]. Therefore, the choice of an ideal sedative agent is crucial. An effective agent should provide comfort, amnesia, and hemodynamic stability and maintain a patent airway with spontaneous respiration. Rapid onset and offset of action are desirable properties. Hemodynamic stress responses to tracheal intubation, including hypertension, tachycardia, and arrhythmias, are mediated by sympathetic stimulation and further justify the need for appropriate sedation [4].

Several agents, such as fentanyl, midazolam, ketamine, propofol, and remifentanil, have been used for facilitating FOI. A review of 14 randomized controlled trials spanning over 30 years highlighted the evidence supporting the use of benzodiazepines, propofol, opioids, alpha-2 adrenoceptor agonists, and ketamine, discussing their efficacy, recommended doses, and limitations in AFOI [5]. Among these, dexmedetomidine, a highly selective alpha-2 adrenoceptor agonist, has gained popularity due to its sedative, anxiolytic, analgesic, anti-sialagogue, and sympatholytic properties, along with its ability to provide anterograde amnesia while maintaining cardiorespiratory stability [6].

Dexmedetomidine can be administered via intravenous, intramuscular, buccal, intranasal, and inhalational routes. Notably, its nebulized form demonstrates favorable bioavailability, with 65% absorption through the nasal mucosa and 82% through the buccal mucosa [6,7]. This pharmacological profile makes nebulized dexmedetomidine an attractive option for non-invasive sedation in AFOI.

## Materials and methods

The study was carried out at Sardar Vallabh Bhai Patel (SVBP) Hospital, affiliated with Lala Lajpat Rai Memorial (LLRM) Medical College, Chaudhary Charan Singh University, Meerut, Uttar Pradesh, India. Data collection spanned 17 months, from May 2023 to September 2024.

Ethical approval and trial registration

This study was conducted after obtaining approval (number SC/-1/2024/3625 dated 16-05-2024) from the Institutional Ethics Committee of LLRM Medical College, Meerut. It was registered with the Clinical Trials Registry of India (registration number: CTRI/2024/07/071652) on July 31, 2024. Throughout the study, strict adherence to ethical principles was maintained to protect patient rights and confidentiality. Written informed consent was obtained from all participants, with the option to withdraw at any stage.

Sample size

In a previous study, the incidence of severe coughing was 15% in the nebulized dexmedetomidine group and 50% in the intravenous dexmedetomidine group. Considering cough score/incidence of severe cough as the primary outcome, the required sample size was calculated for comparison of two proportions with power (1−β) = 0.80 and two-sided α = 0.05. Based on these assumptions, a minimum of 23 patients per group was required, giving a total sample size of 46 patients.

Participants

The study included adult patients of either sex, aged between 18 and 75 years, belonging to the American Society of Anesthesiologists (ASA) physical status I-II, who were scheduled to undergo elective surgical procedures under general anesthesia in the supine position. Patients were excluded if they refused participation, were pregnant, or had a history of cardiac disease, hypertension, atrioventricular block, or heart failure. Those with liver or kidney disease, such as cirrhosis, coagulopathy, or thrombocytopenia, were also excluded. Additional exclusion criteria included chronic alcoholism, drug abuse, psychiatric illness, emergency surgeries, and hemodynamic instability, defined as systolic blood pressure (SBP) <90 mmHg or heart rate (HR) <50/min. Patients who did not meet fasting guidelines (<8 hours for solids and <3 hours for clear liquids), those with respiratory disease, nasal pathology, or a history of epistaxis, and individuals with known allergy or hypersensitivity to study drugs were also not considered eligible.

Randomization and blinding

Patients were randomized into two groups using sealed opaque envelopes to ensure allocation concealment. Study drugs were prepared and administered by an anesthesiologist not involved in outcome assessment. An independent observer, blinded to group assignment, recorded hemodynamic parameters, sedation scores, tolerance, intubation time, recall, discomfort, and adverse events. Patients in the nebulization group (Group N) received dexmedetomidine 1 μg/kg combined with 4 mL of 4% lignocaine via nebulization for four minutes, along with 20 mL of intravenous normal saline infused over 10 minutes. Patients in the intravenous group (Group I) were administered dexmedetomidine 1 μg/kg diluted in 20 mL normal saline intravenously over 10 minutes, along with 4 mL of 4% lignocaine via nebulization for four minutes.

Pre-anesthetic preparation

All patients underwent detailed pre-anesthetic evaluation, including history, physical examination, airway assessment, and routine investigations as per institutional protocol. Pre-medications included oral alprazolam (0.5 mg, the night before surgery), oral ranitidine (150 mg), oral metoclopramide (10 mg) two hours before surgery, and intramuscular glycopyrrolate (0.2 mg) one hour prior. Nasal mucosa was decongested with 0.1% xylometazoline drops. Standard monitoring was applied, and baseline values of HR, mean arterial pressure (MAP), and oxygen saturation (SpO₂) were recorded.

Anesthetic technique

All patients received intravenous midazolam (1 mg) before administration of the study drugs, followed by nebulization and intravenous infusions according to group allocation. Sedation was monitored using the Modified Observer Assessment of Alertness/Sedation (OAA/S) scale, and AFOI was attempted at a score of ≤3; if the score was >3, propofol was administered until adequate sedation was achieved. The more patent nostril was selected, lubricated, and dilated with a nasopharyngeal airway before introducing the fiberscope with a mounted endotracheal tube. Additional anesthesia was provided using the spray-as-you-go (SAYGO) technique with 2% lignocaine. Intubation conditions were assessed by time to intubation, facial grimace score, cough severity, ease of intubation, and hemodynamic parameters (HR, MAP, SpO₂, ECG) recorded at baseline, every two minutes for 10 minutes, and at key intubation stages. Following intubation, anesthesia was induced with fentanyl (2 µg/kg) and vecuronium (0.1 mg/kg). Adverse events such as hypotension, bradycardia, desaturation, trauma, sore throat, dysphagia, or hoarseness were managed and documented. Postoperatively, patients were followed for 24 hours to assess sore throat, discomfort, recall of the procedure, and willingness to undergo AFOI again.

Statistical analysis

Data were analyzed using IBM SPSS Statistics software, version 25 (IBM Corp., Armonk, NY). Continuous variables were expressed as mean ± SD and compared using independent or paired t-tests, as appropriate. Categorical variables were analyzed using the chi-square test. A p-value < 0.05 was considered statistically significant.

## Results

The present study was conducted on 46 patients aged between 18 and 75 years, scheduled for elective surgical procedures requiring nasotracheal intubation. Patients were randomly allocated into two equal groups: Group N (n=23), who received nebulized dexmedetomidine with lidocaine and intravenous saline, and Group I (n=23), who received intravenous dexmedetomidine with lidocaine nebulization.

Demographic characteristics

Baseline demographic parameters were comparable between the groups, with no statistically significant differences in age, gender, weight, or ASA physical status. This ensured that both groups were well-matched and suitable for comparative analysis (Table 1).

**Table 1:** Demographic profile of study groups Group N: nebulization group; Group I: intravenous group; F: female; M: male; SD: standard deviation; ASA: American Society of Anesthesiologists Values are presented as mean ± SD or frequency; an independent Student’s t-test was applied for continuous variables (age, weight), and chi-square (χ²) test for categorical variables (gender, ASA status). p <0.05 was considered statistically significant.

Parameter	Group N (n = 23)	Group I (n = 23)	Test Statistic	p-value
Age (years, mean ± SD)	63.74 ± 8.08	66.13 ± 6.93	t = 1.08	0.287
Gender (M/F)	16 / 7	14 / 9	χ² = 0.09	0.766
Weight (kg, mean ± SD)	63.74 ± 8.08	66.13 ± 6.93	t = 1.08	0.287
ASA I / II	11 / 12	9 / 14	χ² = 0.09	0.766

Sedation and intubation parameters

A highly significant difference was observed in OASS scores, with Group I achieving better sedation (p < 0.05). Similarly, Group I patients required a significantly lower dose of propofol compared to Group N, reflecting superior sedation efficacy (Table 2).

**Table 2:** Comparison of sedation and propofol requirement Group N: nebulization group; Group I: intravenous group; OASS: Observer's Assessment of Alertness/Sedation Scale Values are presented as mean ± SD or frequency (%). Independent Student’s t-test was used for continuous variables (propofol requirement), and chi-square (χ²) test for categorical variables (OASS ≤ 3). p < 0.05 was considered statistically significant. *significant p-value

Parameter	Group N (n = 23)	Group I (n = 23)	Test Statistic	p-value
OASS ≤ 3, n (%)	0 (0.0 %)	23 (100 %)	χ² = 42.46	< 0.001 *
Propofol requirement (mg/kg, Mean ± SD)	4.22 ± 1.31	0.74 ± 1.14	t = 10.09	< 0.001 *

Cough severity

Cough severity differed significantly between the groups. While 21.7% of patients in Group N had severe cough (>5 in sequence) and 30.4% had mild cough, the majority in Group I (69.6%) had no cough. The intergroup difference was highly significant (p < 0.05) (Table 3).

**Table 3:** Comparison of cough severity between groups Group N: nebulization group; Group I: intravenous group

Cough Severity	Group N (n=23)	Group I (n=23)
>5 in sequence	5 (21.7%)	0 (0%)
2–few in sequence	7 (30.4%)	0 (0%)
3–5 in sequence	11 (47.8%)	7 (30.4%)
No cough	0 (0%)	16 (69.6%)

Hemodynamic and other outcomes

Hemodynamic parameters (HR, SBP, diastolic blood pressure (DBP), MAP, and SpO₂) were comparable between the two groups at all measured time points, with no statistically significant differences. The incidence of intraoperative complications was minimal, with only one case of hypotension in Group N. Postoperative complications such as sore throat, dysphagia, or hoarseness of voice were not reported in either group. Recall of procedure did not differ significantly between groups, though Group I reported significantly less discomfort and demonstrated a higher willingness to undergo the same anesthesia again if required.

## Discussion

Anticipated difficult airway management highlights the importance of AFOI, which remains the gold standard in such situations. However, awake nasotracheal FOI is often associated with anxiety, hypertension, and tachycardia if patient preparation is inadequate. Successful AFOI requires patients to be calm, cooperative, and hemodynamically stable, with spontaneous ventilation preserved and minimal airway reactivity [8-12]. Sedatives used for this purpose should be short-acting, titratable, and effective without causing excessive respiratory depression [13-15]. Conscious sedation provides optimal conditions by ensuring a cooperative patient who can follow commands, though over-sedation can result in airway obstruction, hypoventilation, or apnea. Several drugs, including benzodiazepines, propofol, opioids, α2-adrenoceptor agonists, and ketamine, have been used to facilitate AFOI, and dexmedetomidine has gained popularity because of its favorable pharmacological profile [12-15].

In the present study, baseline demographic variables such as age, sex, weight, ASA physical status, and airway parameters were comparable between the groups, and all patients were successfully intubated using the fiberoptic technique. Although intubating conditions were statistically comparable, the intravenous group demonstrated slightly better tolerance. Mondal et al. [8] found better post-intubation scores with intravenous dexmedetomidine compared to fentanyl, supporting our observation that patients receiving intravenous dexmedetomidine were more cooperative (65.22% vs. 39.13% in the nebulization group). Moderate resistance was more frequent in the nebulized group (60.87% vs. 34.78%), which may be attributed to reduced coughing and deeper sedation in the intravenous group.

The mean intubation time was significantly shorter in the intravenous group (5.22 ± 2.26 min) compared to the nebulized group (8.00 ± 2.02 min; p = 0.007). This finding likely reflects better intubating conditions. Sharkawy et al. [9] reported a mean intubation time of 58.9 ± 6.1 seconds using an OAAS score of 2 with 10% lidocaine spray, whereas in our study, we used an OAAS score of 3 and the SAYGO technique with 2% lignocaine, which may have contributed to improved cooperation and orientation. Jarineshin et al. [10] also observed that dexmedetomidine at a dose of 1 μg/kg was more effective than 0.5 μg/kg in attenuating hemodynamic responses to intubation. Similarly, Kumar et al. [6] reported that nebulized dexmedetomidine (1 μg/kg) effectively blunted stress responses without adverse effects, which provided the rationale for choosing this dose for both routes in our study.

Both intravenous and nebulized dexmedetomidine attenuated the pressor response to intubation. Hemodynamic parameters, including HR, SBP, DBP, and MAP, were comparable between the groups at baseline and throughout most intervals. However, at five minutes post intubation, mean SBP values were significantly lower in the intravenous group. Hypotension was noted in one patient (4.35%) in the nebulization group, while no cases occurred in the intravenous group. The slightly higher incidence of hypotension in the nebulization group may have been due to the increased requirement for supplemental propofol.

Patient tolerance, as assessed by the Facial Grimace Score, was superior in the intravenous group. In this group, 52.17% of patients showed no grimace, and 47.83% had only mild grimaces, compared to the nebulization group, where 65.22% had moderate and 21.74% had severe grimacing. Sedation assessed by OAAS also favored intravenous dexmedetomidine. All patients in the nebulized group required additional propofol to achieve OAAS ≤3, whereas all patients in the intravenous group achieved adequate sedation without supplementation. Sharkawy et al. [9] reported similar findings, while Singariya et al. [14], in the Difficult Airway Society guidelines for awake tracheal intubation, emphasized that sedation must be carefully titrated to balance patient comfort with airway safety. Our results support this principle, showing that intravenous dexmedetomidine produced more reliable sedation compared to nebulization.

Interestingly, Zanaty and El Metainy [15] demonstrated that nebulized dexmedetomidine, either alone or combined with ketamine, provided variable sedation in pediatric dental patients. Their findings suggest that patient population and drug combinations strongly influence outcomes, which may explain why our adult cohort showed better results with the intravenous route. In our study, the higher OAAS in the nebulized group likely reflects reduced systemic bioavailability (65%), necessitating higher propofol supplementation. Consequently, propofol use was significantly greater in the nebulized group (4.22 ± 1.31 mg/kg vs. 0.74 ± 1.14 mg/kg; p = 0.00).

Cough severity was significantly lower in the intravenous group, where 69.57% of patients had no cough, compared to the nebulized group, where 21.74% of patients experienced severe coughing (>5 in sequence). Although Gu et al. [11] reported reduced coughing with nebulized dexmedetomidine during bronchoscopy, our findings were the opposite. This difference may be explained by variations in drug delivery, bioavailability, and the type of airway instrumentation. Furthermore, the intravenous group required fewer SAYGO boluses (4.00 ± 1.51 vs. 5.09 ± 1.88; p = 0.04), highlighting better airway anesthesia.

Discomfort during the procedure was also significantly lower in the intravenous group, where nearly half (47.83%) reported no discomfort compared to 13.04% in the nebulized group. Moderate discomfort was much higher in the nebulized group (56.52% vs. 8.7%; p = 0.0014). These findings differ from those of Kumar et al. [6], who observed higher discomfort with ketamine-based regimens in the absence of adequate topical anesthesia. In our study, however, none of the patients experienced pain severe enough to interrupt the procedure.

Postoperative recall was similar in both groups. While 56.52% of patients in the nebulized group and 34.78% in the intravenous group reported no recollection, 65.22% in the intravenous group and 43.48% in the nebulized group had partial recall, which was not statistically significant. This is consistent with the known amnestic effects of dexmedetomidine and the additional sedative effects of propofol in the nebulized group. Sinha et al. [12] also reported higher recall rates in patients receiving dexmedetomidine plus ketamine compared to dexmedetomidine alone, while Tsai et al. [13] found greater recall with dexmedetomidine compared to propofol.

Overall, intravenous dexmedetomidine was associated with improved sedation, reduced propofol and SAYGO requirements, lower cough scores, better intubating conditions, shorter intubation times, less discomfort, and higher patient willingness to undergo repeat AFOI. Nebulized dexmedetomidine, although safe and feasible, was less effective due to lower systemic bioavailability.

Limitations

This study has certain limitations. The inclusion of a broad age group may have obscured age-specific differences in sedation and hemodynamic responses. Being a single-center study limits the generalizability of results. Plasma concentrations of dexmedetomidine were not measured, which could have provided a pharmacokinetic correlation. Finally, the study was conducted in patients with normal airways rather than anticipated difficult airways, limiting direct applicability to higher-risk populations. The use of identical weight-based doses for intravenous and nebulized dexmedetomidine may not reflect true pharmacodynamic equivalence, as nebulized administration has variable bioavailability and delayed onset. The observed lower sedation in the nebulized group may therefore be related to dosing and timing rather than reduced efficacy of the route itself. Although hemodynamic parameters were largely within acceptable clinical limits, the nebulized group showed relatively higher HR and blood pressure values, and one episode of hypotension was observed. Attribution of this event to propofol use remains speculative, and these findings should be interpreted cautiously without implying definitive causal relationships.

## Conclusions

In patients undergoing awake nasotracheal FOI with standardized nebulized lignocaine airway topicalization, both intravenous and nebulized dexmedetomidine (1 μg/kg) effectively facilitated successful intubation with stable hemodynamics. Baseline characteristics were comparable between groups. Intravenous dexmedetomidine provided superior sedation quality, smoother intubating conditions, lower cough and grimace scores, and reduced need for propofol and lignocaine supplementation compared with the nebulized route. Although the nebulized group demonstrated slightly higher HR and blood pressure values during the procedure, overall safety profiles were comparable. Thus, intravenous dexmedetomidine appears to offer more consistent and effective systemic sedation for AFOI when adequate airway topical anesthesia is ensured. Nebulized dexmedetomidine may still have a potential adjunctive role in airway preparation, and its comparatively lower efficacy likely reflects differences in systemic bioavailability and onset characteristics rather than lack of clinical utility.
